# Effect of Online Training during the COVID-19 Quarantine on the Aerobic Capacity of Youth Soccer Players

**DOI:** 10.3390/ijerph18126195

**Published:** 2021-06-08

**Authors:** Paweł Kalinowski, Jakub Myszkowski, Jakub Marynowicz

**Affiliations:** 1 Department of Theory and Methodology of Team Sport Games, Poznań University of Physical Education, 61-871 Poznan, Poland; marynowicz@awf.poznan.pl; 2 Warta Poznan, 61-553 Poznan, Poland; myszkowski.jakub@gmail.com

**Keywords:** football, beep test, cardiovascular, athletic performance, pandemic quarantine

## Abstract

Motor abilities, such as endurance and the optimal level of physical activity, play a fundamental role in football as they are necessary to maintain the high effectiveness of the training process. The aim of this study was the observation of the trend of changes in the level of cardiorespiratory endurance of young football players in a one-year cycle overlapping with the COVID-19 lockdown and an assessment of the impact of the training intervention during home confinement. The participants of the study were 24 young football players. We analysed the results of the study in a one-year training cycle (lockdown from 11 March 2020 to 6 May 2020). The cardiorespiratory endurance was measured using the Multistage 20 m Shuttle Run test—Beep Test. A repeated measures analysis of variance was used in the study. Detailed comparisons were made using Tukey’s HSD test. Statistically significant differences were noted in endurance in a one year cycle: F(5.115) = 22.65; *p* < 0.001; partial Eta-squared = 0.50. An increase in the level of endurance by mean = 179.17 m, SD ± 189.87 m was noted between T1 and T6. After the break caused by the COVID-19 restrictions, a decrease in the level of cardiorespiratory endurance was noted. Only after two training mesocycles was a significant increase in the mean value noted compared to the period before the pandemic (*p* < 0.05). With the negative impact of restrictions in mind, coaches and physiotherapists should exercise caution when planning training, taking into consideration the level of physical activity during the pandemic.

## 1. Introduction

The global COVID-19 pandemic, which was declared on 11 March 2020 by the World Health Organisation [1], placed the sport community in a new reality. For the first time in history, a factor other than a world war put a stop to football competitions over almost the entire world [2]. Many specialists and coaches found themselves in a new, unfamiliar situation. Sports activities of children and youths were completely suspended. In many countries, including Poland, so called lockdown was introduced, which involved restrictions, such as banning the free mobility of children below 16 years of age, contributing to limitations on physical activity.

Researchers have assumed that the restrictions will affect the widely-defined sport development and influence the technical, tactical, motor, and psychological preparation of young athletes [3,4]. Nevertheless, the effect of lockdown on children and youth sport was difficult to predict. In a team discipline, it was no longer possible to work in a group. It was assumed that isolating players from the community would also have mental and social consequences in the long term [3,5]. The suddenly introduced restrictions related to the COVID-19 lockdown resulted in interrupting the continuity of the training cycle, causing changes in the course and organisation of work with players of football academies. The restrictions introduced to protect public health, even though they were indeed necessary, may have had detrimental consequences for mental health and general fitness.

Football is a sport in which aerobic abilities are the basis for performing complex motor activities in changing game conditions [6,7,8]. Highly developed aerobic capacity makes it possible to perform repeated exertions of specific intensity in football during the play. It is assumed that it is key for the performance in difficult conditions during the whole season [9,10]. Aerobic capacity is the ability to continue exercising for a long time and is synonymous with endurance [11,12]. Aerobic capacity most often refers to the maximal lactate steady state, maximum oxygen uptake (VO_2_max) while maintaining equilibrium between the processes of blood lactate accumulation and elimination.

Currently in youth football all over the world, it is assumed that the Maximal Multistage 20 m Shuttle Run Test—Beep Test is one of the most easily available diagnostic tests for the assessment of cardiorespiratory endurance due to its uncomplicated application procedures and the possibility of comparing results [13,14,15]. The Multistage Shuttle Run test as an indirect measure of cardiorespiratory endurance has shown moderate-to-high validity for predicting the maximum oxygen uptake [16]. Intermittent tests are of significant importance for the assessment of the fitness of young players [17,18].

Taking into account comprehensive preparation of players [19], researchers assumed that a total lockdown and a break in training may have both short- and long-term consequences for motor, mental, social, and technical areas [4]. Football clubs were uncertain as to how long the restrictions would last. In the case of a short-term break, one or two weeks without training did not show significant changes in the level of aerobic capacity [20,21]. Attempts were made to find similarities between planning training work during lockdown and breaks in training caused by injury or detraining. 

On the basis of earlier reports, a decrease in the level of motor abilities was expected as early as after five weeks of a break in training [22]. It can be assumed that the circumstances were similar to detraining, that is a training period in order to reduce the physiological and psychological stress of daily training and consequently prepare the athlete’s body for the next training cycle [23,24]. The VO_2_max decreases gradually and proportionally in highly trained and in less highly trained athletes over eight weeks of a break in training [25,26,27]. This phenomenon occurs mainly after intensive training and is perceived as an absence of sporting activity [24]. Detraining is considered in two areas: physical and physiological. 

Changes in the respiratory and cardiovascular systems are classified as the physiological dimension, whereas the decrease in the level of motor abilities, including the endurance, muscular strength, agility, and speed, is part of the physical aspect [28,29]. Detraining is considered in two timelines: shorter than four weeks and longer than four weeks [24,30]. Therefore, taking into account the duration of the restrictions introduced by the government for more than four weeks and the online training applied during the lockdown, we shall consider the training break as long-term detraining. 

The COVID-19 situation differed from anything that had previously occurred in sport, as young players could follow the recommendations of coaches in home conditions only instead of group training and everyday physical activity. Hence, training programmes for athletes were created. With respect to previous publications [24,31], which showed a decrease in the level of training, including the aerobic capacity in football players, due to its link to restrictions and duration of the restrictions, we decided that training measures would be selected through an exercise programme based on functional training aiming to compensate for limited physical activity and attempting to maintain the level of the motor abilities of players. 

The effect of COVID-19 related home confinement on the cardiovascular system of professional footballers [32] was demonstrated; however, the size of the effect of the lockdown and mobility restrictions on the cardiovascular system in young players up to 16 years of age has not been reported so far. There is a lack of information regarding the motor consequences of lockdown for youth soccer players under the age of 16; therefore, comparison of our results with regard to youth athletes is difficult. In a practical aspect, it is interesting to study the effect of replacing group training and everyday physical activity with a preventative training programme carried out in home conditions. Thus far, the effect of lockdown on young players has not been studied.

Therefore, the aim of the study was the observation of the trend of changes in the level of cardiorespiratory endurance of young football players in a one-year cycle including the period of COVID-19-related lockdown and the assessment of the effect of training intervention during home confinement.

## 2. Material and Methods

### 2.1. Participants

The participants of the study were 24 young football players (n = 24; age 14.81, SD 0.96 years, height 172.30 cm, SD 9.03 cm; and weight 59.54 kg, SD 7.83 kg) in the U15 category. Goalkeepers were excluded from the study. The players represented one football club playing in elite youth competition. The study was carried out in Poznań in a one-year training cycle (January 2020–January 2021). During the study, no injuries or SARS CoV-2 infections were noted. None of the players had any PCR and antigen tests for COVID-19. 

All participants expressed their written consent confirmed with a COVID-19 declaration. The participants qualified for the study were players in the under-16 category, without injuries of the motor organs, whose health before and during the study allowed them to participate. The study was carried out as part of monitoring talent identification and development in football in Poland. The study was carried out in accordance with the Helsinki declaration after obtaining an approval of the Bioethics Committee of the Poznań Medical University (decision no. 701/17).

### 2.2. Experimental Approach to the Problem/Procedures/Design

The selection of variables was made on the basis of the literature review. Due to the age of the players and varied individual development, endurance was selected for testing as it develops in a linear manner [33]. Over the period of one year, players took part in the Beep Test six times. The mean levels of endurance were compared in the analysis in six periods of measurement (T1–T6). The assessment of cardiorespiratory endurance and observation of the trend in change of the endurance level were in accordance with the adopted practical recommendations from before the pandemic—namely, at the beginning of the preparatory period, before the competition season, during a break in the competition, and after the season at the end of the year. 

On top of the pre-planned four dates of the assessment of cardiorespiratory endurance and observation of the trend in changes, in accordance with the recommendations and club methodology guidelines, due to an extraordinary pandemic situation, additional dates of endurance tests were added in a week after the 8-week cycle of training intervention (May) and after 4 weeks (June). Two additional dates in May and June were a consequence of the pandemic and aimed to assess the level after the introduction of the training intervention and after the period of training adaptation in normal conditions. Then, after the players achieved a level higher than was measured before the pandemic, the earlier protocol of study was adopted. 

**Figure 1:**
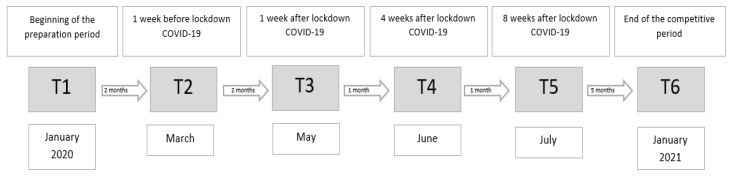
A one-year schedule of cardiorespiratory endurance tests. T 1—January 2020—Start of tests; T 2—March 2020—1 week before the lockdown; T 3—May 2020—1 week after the lockdown; T 4—June 2020—4 weeks after the lockdown; T 5—July 2020—8 weeks after the lockdown; T 6—January 2021—finishing the study after a year-long cycle.

The assessment in July took place according to the study protocol, which had been planned earlier. The diagram showing the duration of individual training blocks and dates of tests is presented in Figure 1. Players ran in groups, in sport (football) footwear. Before the test, there was a 10-min standardised comprehensive warm-up including elements to increase body temperature, muscle activation, and dynamic stretching. All participants completed a standardised warm-up protocol following the RAMP system [34]. The standardised warm-up protocols are detailed in the work of McCubbine et al. [35]. 

The test took place in the same conditions—in a roofed pitch with artificial turf, on a 20 m stretch with one metre safety zone on both sides, on a flat, level, non-slippery ground. Before the test, the rules and form of the test were presented to the players. The test involved running between two points that were 20-m apart in the time marked by a sound signal. The players had to cover each 20-m distance and touch the 20-m line with a foot or cross it during the signal. The initial speed was 8.0 km/h, and after 63 s, it was increased to 9.0 km/h, and then it was increased by 0.5 km/h every 60 s. The test finished when a player decided that he was unable to continue the effort or was unable to maintain the speed of running consistent with the beep. The last level and the number of shuttles completed by the player was recorded and then converted into a distance, which was used in the analysis [6,15].

### 2.3. Description of the Training Intervention

The training intervention focused on maintaining the level of basic motor abilities during the lockdown is described in Table 1.

During the eight week quarantine the players participated in training intervention at home (in their own houses or flats). The training sessions took place online using the ZOOM platform. The ban on outdoor group sports was lifted eight weeks after the announcement of the lockdown. Training intervention (in weeks 1 to 4): An example of a detailed training plan is described in Table 2. All warm-ups were completed on the basis of the RAMP system [34]. Warm-up (15 min) included exercises activating the muscles of lower extremities using resistance bands—mini bands. 

Examples of exercises: standing march, lateral march, forward/backward march, knee raise and hold, knee internal and external rotations (one series, 12 repetitions), dynamic stretching of the body and lower extremities, world greatest stretch, inchworm, tip toes, airplanes, forward lunges, sideways step (one series, 12 repetitions per each side), exercises with elements of central stabilisation of the back, strengthening lower extremities and upper extremities used in football, dead bugs, side planks, glute bridge, and alternating shoulder tap plank. 

All exercises were performed with low intensity, using household equipment, such as 1–2 kg dumbbells or a bottle of the same weight, exercise mat, massage roller, chair, or skipping rope. The intensity of the exercises was monitored continuously and adjusted to the self-perceived level of effort, which was analysed by the coaching staff after each training session using the Borg rating of perceived exertion scale of 1–20 [36]. The Borg scale (RPE) is a method used to monitor exercise intensity in a subjective way. An RPE of 1–9 indicates low intensity, while an RPE of 10–13 and >14 indicates high intensity. RPE is a universal tool, irrespective of locomotor mode and variations in terrain and environmental conditions [37]. Video analysis was used for the assessment of the quality of exercises in real time.

In weeks 5 to 8 of the intervention training volume, the training intensity and the number of repetitions were increased, and co-ordination exercises using an agility ladder with changeable intensity were added (five sets 2 min work (including three exercises of 40 s), recovery between sets = 1.5 min). An example of detailed training programme was described in Table 3. The global training load was calculated by multiplying the training duration (minutes) by the RPE as described by Foster et al. [38]. The session-RPE (sRPE) was the main indicator of load progression. In weeks 5 and 6, the majority of exertions were of medium intensity (60–85% HRmax, RPE 10–13). 

High intensity exercises (90–95% HR max, RPE > 14), which were added in week 6, included running in place with raising knees and exercises with the ladder (for example: double-leg hops, single-leg 2-square hops, alternate-foot ladder sprint, lateral shuffle, and zig-zag crossover shuffle forward and backward). High intensity interval training (HIT) involves repeated short to long bouts of high intensity exercises, interspersed with periods of recovery periods [39] In weeks 7 and 8, a gradual progression from each week was used in the duration of high intensity work, i.e., in weeks 7, exercises with a skipping rope were added alternated with the high intensity. 

High intensity indoor exercises using a skipping rope and planned RPE = 14–16 (3 sets × eight repetitions: 1. Series—1:20 ‘‘work/10” rest; 2. Series—2:15 “work/15” rest; 3. Series—3:10 ‘‘work/20” rest; Recovery between the series: 2 min). In week 8, the number of exercise repetitions was increased to 12. The results of the work with the ladder were monitored using mobile applications (map) and sent to the coaching staff with an RPE assessment. 

After a task performed at home, the players checked their heart rate counting the number of beats over a carotid artery for 10 s. The number of beats was multiplied by 6 to obtain the heart rate per minute. The players were informed about the procedures following the guidelines with respect to fatigue over 14 [40] and the occurrence of COVID-19 symptoms. The intensity of training (regression–progression) was always determined by the same three coaches (the first coach, assistant coach, and strength and conditioning coach) following the assessment of fatigue/heartbeat of the players.

### 2.4. Statistical Analysis

A one-way repeated measured analysis of variance was used. The mean levels of endurance were compared in the analysis in six periods of measurement (T1–T6). The assumption of sphericity typical for a repeated measures analysis of variance was met (Mauchly’s test, *p* > 0.05). To assess the normality of distribution, the W Shapiro–Wilk test was used. The normality of the distribution of endurance was assessed in each of the studied periods. The distribution endurance in four periods (T1 January, T2 March, T4 June, and T5 July) did not differ statistically significantly from a normal distribution. 

The distribution of two measurements (T3 May and T4 June) differed significantly from a normal distribution. The F test with ANOVA is quite resistant to the breach of the condition of normal of distribution [41]. Therefore, in the light of the sphericity assumption being met, the above analysis of variance was used. The effect size was assessed using the partial eta-squared indicator. After receiving a significant value of the F test, detailed comparisons were made using Tukey’s honestly significant difference (HSD) test. The significance level of 0.05 was assumed. The descriptive statistics, including the mean and standard deviation, were presented. Statistical analysis was carried out using Statistica 13.0 software (StatSoft, Krakow, Poland).

## 3. Results

**Figure 2:**
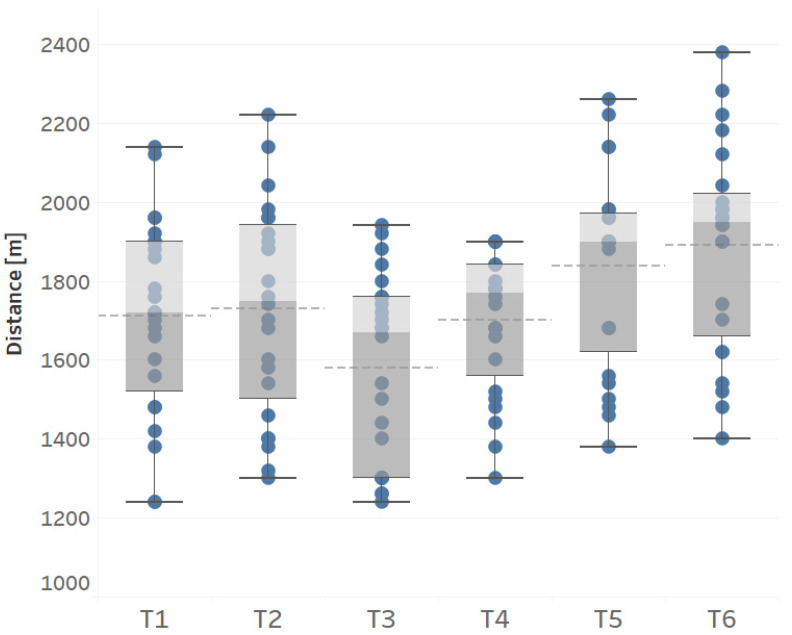
Boxplot for measurements of aerobic capacity in a one-year cycle of young football players. The dotted horizontal line represents the mean of the distribution. The boxes represent the upper and lower quartiles, the whiskers reach the highest and lowest values. Dots represent individual values. T 1—January 2020—Start of tests; T 2—March 2020—1 week before the lockdown; T 3—May 2020—1 week after the lockdown; T 4—June 2020—4 weeks after the lockdown; T 5—July 2020—8 weeks after the lockdown; T 6—January 2021—finishing the study after a year-long cycle.

The collected results are presented in Table 2 and Figure 2. Table 4 shows the analysis of variance, effect size, and descriptive statistics (arithmetic means and standard deviation) of the studied players of Warta Poznań U15 team in a one-year training period on six dates.

Statistically significant differences were noted between measurements in a one-year cycle one-way ANOVA: F(5.115) = 22.65; *p* < 0.001; partial Eta-squared = 0.50 (Table 4). In the third measurement (May) in the second microcycle after the return to normal training following the break caused by COVID-19 restrictions, statistically significant differences were noted compared to all other measurements of endurance made in other periods. After a month-long training mesocycle (June) values similar to the measurement before the pandemic were noted. However, only after two training mesocycles, a significant increase in the mean value was noted compared to the period before the pandemic (*p* < 0.05). Detailed *p* values for comparisons between the measurements and descriptive statistics were presented in Table 4.

## 4. Discussion

The study aimed to analyse the trend in the changes in the level of cardiorespiratory endurance of young football players in a one-year cycle, which included the COVID-19 lockdown, and the assessment of the effect of training intervention during home confinement. The lockdown during the COVID-19 pandemic confirmed the decrease in the level of cardiorespiratory endurance with changes in the traditional training and a decrease in physical activity during the period of home confinement. We assumed that the training interventions in home conditions without optimal physical activity cannot replace organised training. 

While bearing in mind the negative effects of restrictions, coaches and physiotherapists must exercise caution when planning individualised training sessions taking into consideration the optimal level of physical activity in the conditions of the pandemic, in order to minimise the effects of the lockdown or home confinement. Therefore, it may be supposed that training recommendations of at least 200–400 min of aerobic exercise per week [42] and two resistance training sessions are sufficient during quarantine [43] only in the case of maintaining the optimal level of everyday physical activity. 

According to the latest guidelines of the WHO [44], during the pandemic, children and youths should participate in 60 min of moderate activity per day and at least three times per week of high intensity aerobic exercises to strengthen bones and muscles [44]. Hence, with government restrictions, it could be assumed that, in the case of decreased physical activity, the volume and intensity of training should be increased in order to compensate for the energy balance. The consequences of the COVID-19 pandemic among young players are both short- and long-term [2]. The measurements of aerobic endurance in a one-year cycle showed interesting results after the lockdown. An initial higher level of respiratory and circulatory endurance was a predictor of higher decreases in the level. 

Multistage fitness test performance improves linearly with age [45]. This study showed that the linear increase in the level of endurance in the studied group was inhibited. The return to the pre-lockdown level took eight weeks for the studied group. This shows the negative impact of COVID-19 home confinement on the changes in the trend of the endurance level. In the case of traditional endurance training, an increase in the maximum oxygen consumption was noted after eight weeks in 102 Danish male and female football players [46]. 

However, as a result of the introduction of an 8-week lockdown, an expected linear increase in the level of endurance in young players in a one-year cycle was not noted. The results of the study that was carried out in March 2020, a week before the lockdown, did not differ statistically significantly from the results of the study that took place in June 2020, four weeks after the lockdown. These results did not match the results of studies carried out in normal conditions by Śliwowski et al. [47] who studied junior players from Lech Poznań using the Multistage Shuttle Run (MST). 

In their study on two dates over the eight weeks of the preparatory period, the authors demonstrated favourable adaptive changes in the studied physiological indicators, such as aerobic capacity and anaerobic threshold, between two dates of the measurements—before the preparatory period and after the end of the preparatory period. Thus, the unfavourable effect of the lockdown period can be considered. When attempting to make comparisons, we should bear in mind that the COVID-19 situation occurred for the first time and cannot be directly compared to any earlier event [4]. 

Although the periods of breaks in training and detraining occur regularly in youth sports, the restrictions introduced by the Polish government with a complete ban on mobility for persons below the age of 16 took place for the first time in the history of football. Previous studies provide information about linear increases in the level of physical endurance in a one-year training cycle [46]. However, the COVID-19 quarantine resulted in a decrease in the level of endurance of the studied players in a significant way after the period of pandemic related restrictions from March to May 2020.

Thus far, authors have investigated the mental and social consequences [3,5] regarding motor quarantine for professional athletes [31]. Albuquerque et al. [32] showed that COVID-19-related restrictions and quarantine had adverse effects on professional soccer players’ Yo-Yo Intermittent Recovery Test performance; however, the level of relative distance referred to differences between the pre- versus post-COVID-19 quarantine Yo-Yo test results. Our study of young athletes did not confirm the reports of a study on an adult group. This may be due to the limitations of physical activity for young people under the age of 16. 

Our review of the literature established that factors, such as the quality of sleep and diet, can also contribute to a decrease in the level of endurance. The studied players did not change their nutritional habits and did not consume caffeine [14,48]. During the lockdown all players declared that they slept for 7–10 h per night [49]. In spite of this, the results of our study did not confirm the earlier study of Azizi et al. [14], who noted no changes in the level of physical efficiency following the change in the type of training in the course of a 40 day Ramadan observance. 

Our study showed an unfavourable effect of the lockdown on the cardiovascular system in the studied group. These results may be indicative that other factors co-occurring during home confinement may also contribute to the significant fall in the level of endurance. The results of the study of 24 young football players in Poland were consistent with the results of Mujika and Padilla [30], who demonstrated a decrease in the level of endurance due to the absence of an optimal training load.

These reports showed that virtually all players, regardless of their initial level, noted a decrease in the level of endurance during the lockdown. A higher level of VO2max causes faster phosphocreatine (PCr) recovery kinetics [33], which is necessary for normal sports training. It is known that a short-term break of one or two weeks without training does not cause significant changes in the level of aerobic capacity [20,21], whereas a decrease in motor abilities can be expected as soon as after five weeks of a break in training [22]. After eight weeks of detraining, a decrease in VO2max was noted, which was maintained at the higher level in more highly trained athletes and at the lower level in less highly trained athletes [26,27]. The training intervention was intended to replace the lack of group training and decreased physical activity.

While we could make a comparison to the detraining situation and accept it as natural in such a long period without training, considering the preventative programme of training intervention at home, the size of the decrease was unexpected. This study shows the short-term consequences of the COVID-19 pandemic, in particular the lack of physical activity, mobility, or even recreational running or cycling. Another interesting observation shown by the study in a one-year perspective is the time necessary to return to the earlier level after an eight week home confinement, which was two full training mesocyles. 

It should be considered whether this permanently inhibited harmonious development of young players and, if yes, to what extent. It is likely not possible to determine this now. It is difficult to directly compare the situation that occurred with a control group because no one has planned such an intervention before. Similarly it can be assumed that the absence of teamwork, contact with football, and participation in organised training for the period of over 4 weeks will have even greater impact. In all probability, the consequences will became apparent after the lockdown and the global pandemic and will be felt for a few years, particularly in relation to children and youth sports. 

It can be supposed that COVID-19, despite everything, affected adult men to a smaller extent that youths, due to the fact that preparation of the body to exertion after the detraining period for adults will be focused mainly on returning to the previous level of motor abilities. In case of children and youths, we are dealing with both the inhibition in development of motor abilities and decrease in the possibility of learning technical and tactical skills. Therefore, it may be assumed that in 2020, players in youth and children categories were facing a more difficult situation compared to their peers in the previous decade. It also is doubtful that the long-term stress and anxiety caused by the restrictions will have a positive effect on anyone [3,4,5]. Unfortunately, over the years, we will experience new consequences of these difficult times.

### 4.1. Practical Implications

The data concerning the decrease in the level of cardiorespiratory endurance may help in the development of training interventions after a detraining period in the future. The conclusions of the study concerning other factors contributing to such a marked decrease showed a significant influence of the everyday physical activity carried out outside training sessions. According to the recommendations of the WHO [44], home training should include 60 min of moderate activity per day and high intensity exertions at least three times per week. 

These days, with constant hurry and widespread car use, it should be worthwhile to establish the minimum physical activity necessary to achieve high results in sports. Our study shows not only to the coaching staff but also to parents the importance of everyday physical activity for the development of children. In times of the universal use of computers and cars, it appears reasonable to develop training programmes, including not only training volume and size but also a specific minimum level of physical activity outside training sessions, which will compensate for the time spent in front of the computer.

### 4.2. Limitations

A limitation of the study is that it focused only on the study the level of cardiorespiratory endurance without other motor abilities. Another limitation is the absence of the possibility of precise comparisons of the volume of work performed during the training intervention with the volume of organised training work and the volume of decreased physical activity caused by government restrictions on mobility in Poland due to varied living conditions.

## Figures and Tables

**Table 1:** Training intervention of the studied players during the lockdown in Poland (11 March 2020–6 May 2020).

Week	Number of Training Sessions per Week	Duration of a Training Session	Aim	Training Measures	Methods of Control
1–3	4/week	60 min	Stability/ROM Low intensity	Individual training	Video analysis, RPE
4	5/week	60 min	Stability/ROM Low intensity	Individual training	Video analysis, RPE
5–6	6/week	60–75 min	Stability/aerobic endurance High intensity	Running/individual training	Distance, time, map, RPE
7–8	6/week	60–75 min	Stability/aerobic endurance High intensity	Running/individual training	Distance, time, map, RPE

RPE— Borg scale.

**Table 2:** An example of a detailed training plan (weeks 1–4).

Part of the Training Unit	Exercises/Goal/Execution (Volume)
Warm-up (15min)	Running in place (2′) Raising knees (12 rep) Forward shoulder circulation (20″) Back arm circulation (20″) Core activation (2 exercises) Glute activation (2 × 12 rep each leg) Ground mobility (3 exercises) Dynamic stretching (12 rep × 7 exercises) A-skip (10″) Butt kicks (10″) Running rhythm in place (10″)
Main Part (35 min)	Balance and coordination exercises (2 ex.) Lower body pull exercises—hip and knee dominant (2 ex.) Upper body push and pull exercises (2 ex.) Hip abductor and adductor exercises (2 ex.) Core stability—lumbo-pelvic control
Cool Down (10min)	Foam rolling (10′)

**Table 3:** An example of a detailed training programme (weeks 5–8).

Part of the Training Unit	Exercises/Goal/Execution (Volume)
Warm-up (15 min)	Running in place (2′) Raising knees (12 rep) Cross step (interlace) (12 rep) Forward shoulder circulation (30″) Back arm circulation (30″) Core activation (2 exercises) Glute activation with a mini band (2 × 12 rep each side) Ground mobility (3 exercises) Dynamic stretching (12 rep × 7 exercises) A-skip (10″) Butt kicks (10″) Running rhythm in place (10″)
Main Part (45 min)	Core stability—lumbo-pelvic control (3 ex.) Exercises with bands, global patterns (2 ex.) Balance and coordination exercises (2 ex.) Running in place <14 RPE: Running in place/Jump rope skipping (5 × 2 min of work, 1.5 min (passive) break RPE 15–18)
Cool Down (10 min)	Foam rolling (10′)

**Table 4:** Comparison of the mean cardiorespiratory endurance among the six time periods. Results of one-way repeated measure analysis of variance and detailed comparison between the means for various period using Huckey’s HSD test.

Measurements	Mean Cardiorespiratory Endurance among the Six Time Periods						
	Months	T 1 M = 1710.8 (m) ±294.52 (m)	T 2 M = 1730.8 (m) ±266.50 (m)	T 3 M = 1580.0 (m) ±237.38 (m)	T 4 M = 1701.7 (m) ±179.75 (m)	T 5 M = 1837.5 (m) ±248.14 (m)	T 6 M = 1890.0 (m) ±262.16 (m)
T 1	January 2020		-	-	-	-	-
**T 2**	March 2020	*p* > 0.05		-	-	-	-
**T 3**	May 2020	***p*** **< 0.01**	***p*** **< 0.001**		-	-	
T 4	June 2020	*p* > 0.05	*p* > 0.05	***p*** **< 0.01**		-	-
T 5	July 2020	***p*** **< 0.01**	***p*** **< 0.05**	***p*** **< 0.001**	***p*** **< 0.001**		-
T 6	January 2021	***p*** **< 0.001**	***p*** **< 0.001**	***p*** **< 0.001**	***p*** **< 0.001**	*p* > 0.05	

SD ± standard deviation; M—mean; T 1—January 2020—Start of tests; T 2—March 2020—1 week before the lockdown; T 3—May 2020—1 week after the lockdown; T 4—June 2020—4 weeks after the lockdown; T 5—July 2020—8 weeks after the lockdown; and T 6—January 2021—finishing the study after a year-long cycle.

## Data Availability

The data presented in this study are available on request from the corresponding author.
